# Retraction: Genome-Wide Analysis of the Dof Transcription Factor Gene Family Reveals Soybean-Specific Duplicable and Functional Characteristics

**DOI:** 10.1371/journal.pone.0167019

**Published:** 2016-11-17

**Authors:** Yong Guo, Li-Juan Qiu, PLOS ONE Editors

The authors acknowledge that there are overlaps between the text of this article and the following previously published work, which was not cited:

Hu R, Guang Q, Yingzhen K, Dejing K, Gao Q and Zhou G (2010) Comprehensive Analysis of NAC Domain Transcription Factor Gene Family in Populus trichocarpa. BMC Plant Biology. DOI: 10.1186/1471-2229-10-14

The authors apologize for the overlap in text.

The *PLOS ONE* Editors have also identified overlaps between the text of this article and a number of previously published works. Text was duplicated verbatim or with minor modifications from the sources listed below:

Genome wide identification of Dof transcription factor gene family in sorghum and its comparative phylogenetic analysis with rice and Arabidopsis. Molecular Biology Reports. DOI: 10.1007/s11033-010-0650-9 (cited as Reference 11 in the published article)

Dof Domain Proteins: Plant-Specific Transcription Factors Associated with Diverse Phenomena Unique to Plants. Plant & Cell Physiology. DOI: 10.1093/pcp/pch055 (cited as Reference 14 in the published article)

Genome-wide Analysis of Plant-specific Dof Transcription Factor Family in Tomato. Journal of Integrative Plant Biology. DOI: 10.1111/jipb.12043 (cited as Reference 57 in the published article)

Identification of cis-regulatory elements specific for different types of reactive oxygen species in Arabidopsis thaliana. Gene. DOI: 10.1016/j.gene.2012.02.035 (uncited in the published article)

The family of DOF transcription factors in Brachypodium distachyon: phylogenetic comparison with rice and barley DOFs and expression profiling. BMC Plant Biology. DOI: 10.1186/1471-2229-12-202 (cited as Reference 54 in the published article)

In light of the concerns identified, the authors and the *PLOS ONE* Editors retract this article.

## References

[1] Guo Y, Qiu L-J (2013) Genome-Wide Analysis of the Dof Transcription Factor Gene Family Reveals Soybean-Specific Duplicable and Functional Characteristics. PLoS ONE 8(9): e76809 doi:10.1371/journal.pone.0076809 24098807 10.1371/journal.pone.0076809 PMC3786956

